# Chemotypic and Seasonal Variations in Essential Oils from *Mespilodaphne cymbarum* (Kunth) Trofimov and Their Antibacterial and Antibiofilm Activities

**DOI:** 10.3390/plants14131939

**Published:** 2025-06-24

**Authors:** Amanda Galdi Boaretto, Darlene Gris, Jéssica Scherer, Katyuce Souza Farias, Jean Carlo Quadros, Alexandre José Macedo, Carlos Alexandre Carollo, Denise Brentan Silva

**Affiliations:** 1 Instituto de Biociências, Universidade Federal de Mato Grosso do Sul, Cidade Universitária, Campo Grande 79070-900, Mato Grosso do Sul, Brazil; amanda.boaretto@ufms.br; 2 Laboratório de Produtos Naturais e Espectrometria de Massas (LaPNEM), Faculdade de Ciências Farmacêuticas, Alimentação e Nutrição (FACFAN), Universidade Federal de Mato Grosso do Sul, Cidade Universitária, Campo Grande 79070-900, Mato Grosso do Sul, Brazil; katyuce.farias@gmail.com (K.S.F.); carlos.carollo@ufms.br (C.A.C.); 3 Instituto de Desenvolvimento Sustentável Mamirauá, Estrada do Bexiga, Tefé 69553-225, Amazonas, Brazil; darlene.gris@mamiraua.org.br (D.G.); jeancdq@gmail.com (J.C.Q.); 4 Laboratório de Biofilmes e Diversidade Microbiana, Faculdade de Farmácia e Centro de Biotecnologia, Universidade Federal do Rio Grande do Sul, Porto Alegre 91501-970, Rio Grande do Sul, Brazil; jessicscherer@gmail.com (J.S.); alexandre.jose.macedo@gmail.com (A.J.M.)

**Keywords:** bark, leaves, fruit, flooding, *Ocotea cymbarum*, Gram-positive, louro-inamuí

## Abstract

This study investigated the essential oils (EOs) from leaf, bark, and fruit of *Mespilodaphne cymbarum* (Kunth) Trofimov (Lauraceae), focusing on their chemical composition and antimicrobial and antibiofilm activities. EOs were extracted from plants collected in the Amazon during dry and flood seasons and analyzed by gas chromatography–mass spectrometry. Although chemical differences were evident among plant organs and chemotypes, the influence of seasonality was not pronounced. Fruit EO was dominated by α- and β-santalene and limonene. Bark EO was rich in phenylpropanoids, including methyl eugenol, myristicin, and elemicin. Leaf EO showed the greatest metabolic diversity, with chemotype-specific variations. Leaf and bark EOs demonstrated superior antibacterial and antibiofilm activities compared to fruit EO, especially against Gram-positive bacteria such as *Staphylococcus epidermidis*, *Staphylococcus aureus*, and *Micrococcus luteus*. Chemotype-1 leaf and bark EOs inhibited *S. epidermidis* biofilm formation, while chemotype-2 reduced bacterial growth. The leaf EOs from both chemotypes reduced bacterial growth against *S. aureus*, and bark EO decreased biofilm formation. All leaf and bark EOs showed antibiofilm activity against *M. luteus*. These findings highlight the potential of *M. cymbarum* EOs as natural sources of bioactive compounds and emphasize the importance of chemotype and plant organ selection for optimized applications.

## 1. Introduction

The botanical family Lauraceae Juss. is recognized as a source of essential oils (EOs) with numerous biological properties and industrial applications, such as cinnamon (*Cinnamomum* spp.), laurel (*Laurus nobilis*), and rosewood (*Aniba rosaeodora*) [1,2]. Predominantly distributed in tropical regions, Lauraceae encompasses approximately 55 genera and 2500–3500 species [3]. Among these, the *Ocotea* Aubl. complex stands out, comprising around 16 genera and 700 species, many of which hold economic and biological importance, including *Nectandra*, *Aniba*, *Licaria*, and *Ocotea* [3]. Genetic analyses based on nuclear internal transcribed spacer (ITS) sequences led to the reinstatement of the genus *Mespilodaphne* Nees & Mart. and the reclassification of *Ocotea cymbarum* Kunth as *Mespilodaphne cymbarum* (Kunth) Trofimov [4]. The genus *Mespilodaphne* includes eight species, distributed across South and Central America, as well as in tropical forests of the Antilles at elevations of up to 2000 m [4].

*Mespilodaphne cymbarum* is popularly known as “louro-inamuí” or “louro-inhamuí” and can be found throughout the Amazon Region with occurrences in Brazil, Colombia, Venezuela, and Guyana [5]. This species is commonly observed in várzea forests, such as the banks of the Solimões River, where it is subjected to periodic flooding [5,6]. Due to its valuable wood, *M. cymbarum* has been exploited for timber [6,7]. The wood, bark, and leaves of *M. cymbarum* are known to contain neolignans and have also been reported as sources of essential oils (EOs) [8,9,10]. The neolignan burchelin, isolated from the bark of *M. cymbarum*, demonstrated potential in vitro activity against both the epimastigote and trypomastigote forms of *Trypanosoma cruzi* [9]. Another neolignan, biseugenol, was isolated from leaves and showed anti-inflammatory activity, as well as inhibition of angiogenesis and fibrogenesis [10].

Research on the EOs of *M. cymbarum* has focused on the bark and trunk wood [7,8,11,12,13]. Shukis and Wachs [14] reported the presence of safrole in this species, but studies employing modern analytical techniques failed to confirm this occurrence [7,11,12]. In addition, the metabolites α-phellandrene, *p*-cymene, and α-pinene were reported from EO of trunk wood *M. cymbarum* [8]. In contrast, EOs of sassafras from wood/bark cited as major components the monoterpenes α-terpineol (34.9%), α-pinene (18.5%), fenchol (6.3%), and borneol (6.2%) [11]. Recently, the EO from sapwood and heartwood of *M. cymbarum* was analyzed by SPME and the volatiles with higher relative area percentage were α-copaene (17%), 1,8-cineole (11%), *trans*-calamene (7.4%), α-calacorene (6.4%), and δ-cadinene (5.5%) [7]. Meanwhile, in the EO of *M. cymbarum* bark, α-selinene (26%), δ-cadinene (19%), terpinen-4-ol (9%), and α-cadinol (6.2%) were described as major components [12].

Residents of Amazonian communities have reported that *M. cymbarum* produces aromatic oils in multiple plant organs, highlighting its potential for sustainable use by local populations. Given the lack of data on the chemical and biological properties of essential oils from *M. cymbarum* leaves and fruits, this study provides a detailed characterization of oils from different plant organs and their antimicrobial and antibiofilm activities against six pathogenic bacterial strains. Samples were collected during both dry and flooding seasons in a seasonally flooded forest (várzea) of the central Amazon to explore potential seasonal and chemotypic variations.

## 2. Results and Discussion

### 2.1. Yields of the Essential Oils (EOs) from Bark, Leaf and Fruit of Mespilodaphne cymbarum

Our study reported for the first time the yield and chemical composition of the essential oils (EOs) from leaf (Le) and fruit (Fr) of *Mespilodaphne cymbarum* (Table 1). The highest yield was recorded for fruit EO (3.6%). Two distinct chemotypes were identified based on pilot studies using solid phase microextraction (SPME) and GC-MS on leaves from 14 individuals (Figure S1, Supporting Information). According to the SPME analysis, we detected several volatiles distinguishing the chemotypes, with the most prominent differences observed in the levels of α- and β-santalene, which were higher in chemotype-1, whereas chemotype-2 exhibited greater concentrations of β-caryophyllene and unknown V82. Consequently, the individuals were grouped accordingly before EO extraction, resulting in four experimental groups that reflected both seasonal and chemotypic variation: Le-F-1 (leaves from the flooding season of chemotype-1), Le-D-1 (leaves from the dry season of chemotype-1), Le-F-2 (leaves from the flooding season of chemotype-2), and Le-D-2 (leaves from the dry season of chemotype-2).

The EOs from the leaves showed similar yields from the two chemotypes (1 and 2) and the dry and flooding seasons. The essential oils from leaves collected in Le-D-1 and Le-F-1 yielded 0.91%, while Le-D-2 and Le-F-2 returned 1.2 and 0.93%, respectively. The bark EOs resulted in lower yield than the other parts, showing yields of 0.6% for Ba-D-1 and 0.5% for Ba-F-1. These results for bark surpassed the bark EO yield (0.1%) previously reported for the same species [12].

Compared to other species within the Lauraceae family, particularly in the genus *Ocotea* and closely related taxa, *M. cymbarum* exhibits remarkably high essential oil yields, especially from its fruits (3.6%) and leaves (~1%). These values are substantially higher than those reported for several *Ocotea* species, where leaf yields often remain below 1% and bark or stem oils are even more limited [13,15,16,17,18,19]. Moreover, the consistency in leaf EO yields across both dry and flooding seasons in *M. cymbarum* is noteworthy, suggesting a level of phenotypic or physiological stability that could be advantageous for sustainable harvesting and commercial exploitation.

### 2.2. Chemical Composition and Statistical Analysis of the Essential Oils (EOs) from Bark, Leaves and Fruits of Mespilodaphne cymbarum

**Figure 1:**
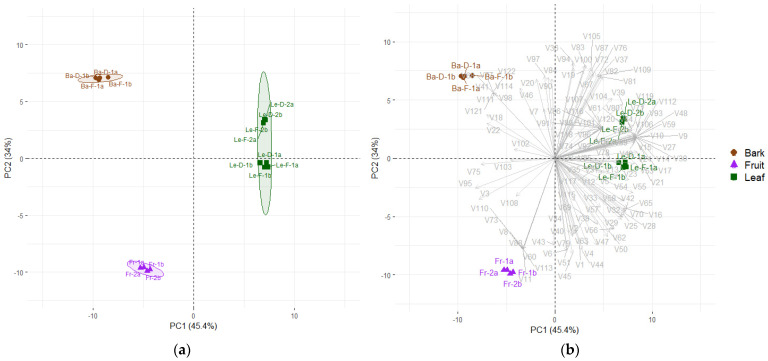
Principal Component Analysis (PCA) of the essential oils (EOs) from leaves, bark and fruits from *Mespilodaphne cymbarum*. (**a**) Score plot illustrating three distinct groups of bark (Ba), fruit (Fr) and leaf (Le) (PERMANOVA, R^2^ = 0.9078; *p* < 0.001); (**b**) biplot exhibiting all volatiles from the EOs.

From the EOs, we detected 122 metabolites across different plant organs (Table 1). Principal components analysis (PCA) was performed using 16 samples, distributed as follows: eight replicates from leaves (Le)—comprising two analytical replicates for each leaf EOs group (Le-D-1, Le-F-1, Le-D-2, Le-F-2), four from bark—two analytical replicates per season (Ba-D-1; Ba-F-1), and four analytical replicates from fruit EO, obtained from two hydrodistillation extractions (Figure 1a). The first two principal components, PC1 (45.4%) and PC2 (34%), explained 79.4% of the total variance, revealing three distinct groups (Figure 1a; PERMANOVA, R^2^ = 0.9078, *p* < 0.001). PC1 clearly separated leaf EOs from bark and fruit EOs (Figure 1). The metabolites contributing to this separation were V9, V10, V14, V15, V17, V24, V26, V27, V30, V48, V59, V89, V93, V106, V112, V117, V119, and V120—detected exclusively in leaves—and V13, V21, V23, V49, V53, V54, and V55 that were higher in this group. In contrast, V3, V22, V75, and V95 were the key volatile compounds contributing to the separation of fruit and bark EOs from leaf EOs along PC1 (Figure 1b).

Leaf EOs exhibited the highest metabolite diversity, containing 105 volatiles, with 34 metabolites detected exclusively in these samples. Sesquiterpene hydrocarbons were the major constituents of all leaf EOs, accounting for approximately 39% in samples from the flooding season, and around 30% in those from the dry season (Table 1). Chemotype-1 contained higher amounts of oxygenated monoterpenes, with 12.6% (Le-F-1) and 15.3% (Le-D-1), as well as monoterpene hydrocarbons at about 7.6% (Le-F-1) and 8.3% (Le-D-1). On the other hand, chemotype-2 exhibited a higher proportion of oxygenated sesquiterpenes, reaching 14.9% (Le-F-2) and 15.8% (Le-D-2). The complex volatile profile of the leaf EOs resulted in the coelution of several metabolites, which made their identification particularly challenging.

**Figure 2:**
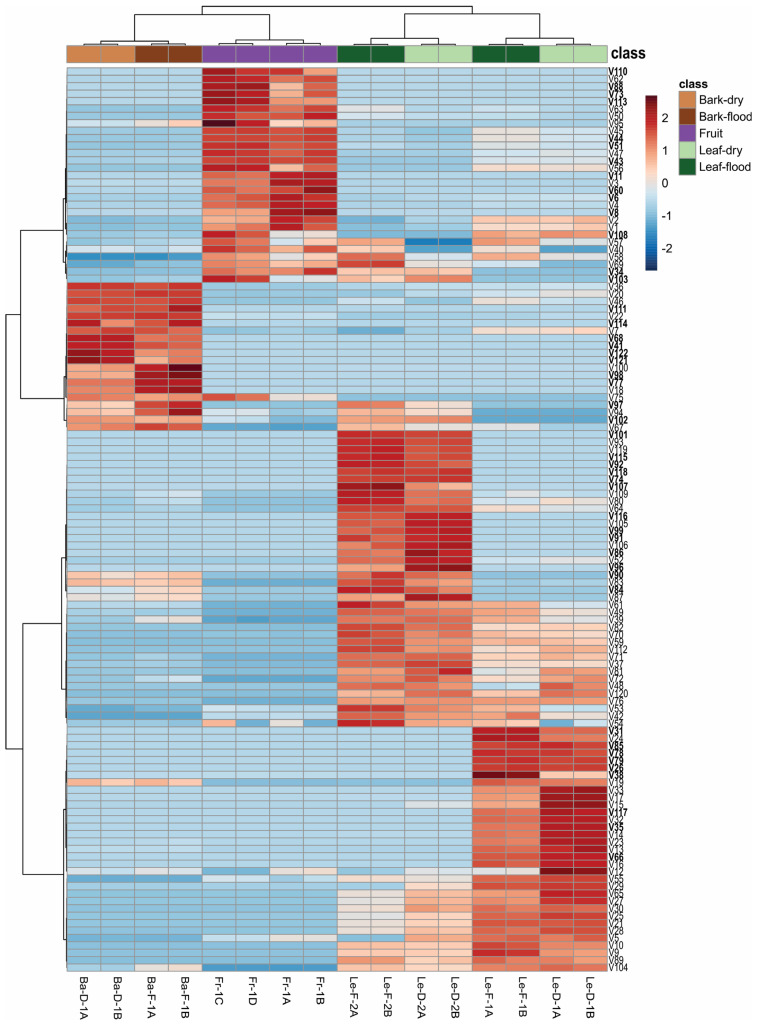
Heatmap and Hierarchical Clustering Analysis (HCA) showing the relative area (%) of each volatile compound of the essential oils (EOs) from bark (Ba), leaf (Le) and fruit (Fr) of *Mespilodaphne cymbarum*. Bark and leaf samples were collected in both the dry (D) and flooding (F) seasons. A, B, C, and D represent the analytical replicates and 1 and 2 are the chemotypes. Metabolites highlighted in bold represent the principal compound of each cluster, including those found exclusively in specific samples or present at a higher relative abundance.

The two chemotypes analyzed in our study showed qualitative differences in their leaf EOs (Table 1; Figure 2). Leaf EOs from chemotype-1 contained 10 metabolites (V26, V31, V35, V38, V66, V78, V79, V85, V108, and V117) that were not detected in chemotype-2. Conversely, chemotype-2 presented 17 metabolites (V34, V74, V84, V86, V90, V91, V92, V96, V97, V99 V101, V102, V103, V107, V115, V116, and V118) that were not found in chemotype-1 (Figure S2, Supporting Information). However, some of these compounds from both chemotypes also occurred in fruit or bark EOs (Table 1).

The EOs from leaves of chemotype-1 presented as major metabolites, showing relative areas higher than 5% for the volatiles β-caryophyllene (V42) (Le-F-1 = 7.9%; Le-D-1 = 4.7%), α-santalene (V43) (Le-F-1 = 6.2%; Le-D-1 = 6.3%), unknown *m*/*z* 234 (V82) (Le-F-1 = 5.4%; Le-D-1 = 5.9%), and cryptone (V21) (approximately 5% in both seasons). Chemotype-2 presented the metabolites unknown *m*/*z* 234 (V82) (Le-F-2 = 11%; Le-D-2 = 9.8%), β-caryophyllene (V42) (Le-F-2 = 9%; Le-D-2 = 7.6%), and δ-cadinene (V67) (Le-F-2 = 5.1%; Le-D-2 = 3.6%), as major volatiles (>5%). Although the compound unknown *m*/*z* 234 (V82) is present at high intensity, its fragmentation pattern does not match any metabolites available in the databases or compounds described in the literature. Therefore, its identification would require isolation and further chemical analyses to characterize its molecular structure. However, such characterization is beyond the scope of this study.

Studies examining the chemical composition of EOs from *Ocotea* and *Mespilodaphne* have identified monoterpenoids, sesquiterpenoids, and phenylpropanoids as the major classes of volatiles [13,16,20]. Among these, sesquiterpenes have been reported as the predominant class in several *Ocotea* species [21,22]. The leaf EO of *Mespilodaphne quixos* (=*Ocotea quixos*) from Ecuador was mainly composed of sesquiterpenes (35.6%), oxygenated monoterpenes (24.8%), and monoterpene hydrocarbons (21.7%), with β-caryophyllene (15.1%), cinnamyl acetate (11.4%), and sabinene (7.6%), as the main volatiles [23]. From *M. quixos* leaf EOs, collected in the Amazonian region of Pastaza (Ecuador), it was reported that oxygenated monoterpenes were the most abundant compounds, with 1,8-cineole (39.1%) and α-terpineol (7.6%) as the primary constituents, followed by hydrocarbon monoterpenes such as sabinene (6.46%), α-pinene (6.3%), and *p*-cymene (6.1%) [24]. Other commonly detected metabolites in *M. quixos* leaf EOs include the aromatics *trans*-cinnamaldehyde, *trans*-methyl cinnamate, and *trans*-cinnamyl acetate [19,25,26]. In *Mespilodaphne veraguensis* (=*Ocotea veraguensis*), oxygenated sesquiterpenes were the predominant class in leaf EO (58.8%), with bulnesol (29.5%), and spathulenol (8.5%) as the main constituents, followed by monoterpene hydrocarbons (27.5%), primarily *p*-cymene (19.8%) [20]. Chaverri et al. [16] observed in the leaf EO of *Mespilodaphne morae* (=*Ocotea morae*) a high percentage of monoterpenes, including β-pinene (17.5%), α-pinene (10.4%), and 1,8-cineole (7.3%); and sesquiterpenes, such as bicyclogermacrene (8.8%), germacrene D (7.5%), and β-caryophyllene (7.1%).

The pronounced chemical variability observed among *Mespilodaphne* and *Ocotea* species, ranging from sesquiterpene to monoterpene dominant profiles, underscores the influence of taxonomic and geographic factors. Notably, β-caryophyllene frequently emerges as a predominant constituent across several species [13,20,23]. This metabolite was detected in both chemotypes. Conversely, the presence of α-santalene (V43) in higher amounts in chemotype-1 was relevant to distinguish this chemotype, since it was detected only in trace amounts in chemotype-2. The presence of α- and β-santalene is not commonly reported in this genus [13]. However, these compounds were detected at low concentrations—approximately 0.36 and 0.11%, respectively—in the flower calyces of *M. quixos* [27].

Although cryptone (V21) and δ-cadinene (V67) were present in leaf EOs from both chemotypes, cryptone (V21) was greater in chemotype-1, while δ-cadinene (V67) was more abundant in chemotype-2. The occurrence of distinct chemotypes may be influenced by genotypic factors as well as abiotic and/or biotic pressures [28,29]. In *M. quixos*, the presence of two distinct chemotypes, a *trans*-methyl cinnamate chemotype and *trans*-caryophyllene and *trans*-cinnamyl acetate chemotype, were determined, which were influenced by environmental conditions, such as soil composition, heights and shade percentage [26]. Similarly, diverse chemotypes in *Nectandra megapotamica* were recorded regarding the volatile profile from specimens collected in different geographic locations, but other compound classes, such as non-volatile phenolics, were similar within populations [30].

In the EO of fruits, we found 66 compounds, predominantly sesquiterpenes hydrocarbons (65.1%), such as α-santalene (V43) (26.4%), β-santalene (V51) (12.0%), and *trans*-α-bergamotene (V44) (8.18%). These compounds are the same as those found in the sandalwood oil extracted from *Santalum album*, which are extensively used by the perfumery and fragrance industries, presenting high economic value [31]. Santalenes and santalols have been detected in 32 species, including the Lauraceae *Cinnamomum camphora*, but in a low concentration of about 2.7% [31]. Therefore, in addition to displaying a valuable sesquiterpene profile, the fruit EO of *M. cymbarum* also showed a high yield (3.6%), highlighting the potential of this resource. Furthermore, we used the fruit pulp, which allowed us to obtain the material while preserving the seeds, enabling a sustainable use of this species. In addition to the potential applications of its essential oil, our results suggest that this species may also be a valuable source for the discovery of novel enzymes involved in the biosynthesis of santalenes and santalols.

Monoterpene hydrocarbons were also abundant in fruit EO, accounting for approximately 28% with limonene (V6) (19.32%) as a prevalent metabolite (Figure 2). Metabolites distinguishing the fruit EOs in the PCA included V8, V11, V60, V73, V88, V110, and V113, which were exclusively found in this sample, while V1, V3, V6, V43, V45, and V51 were present at higher percentages in fruit EO (Table 1; Figure 1b).

Studies analyzing the EO of fruits from *Ocotea* and *Mespilodaphne* are scarce [15,32]. The fruit EO from *Ocotea duckei* also exhibited high amounts of limonene (30.1%), with other major volatiles being α-pinene (12.2%) and β-pinene (9.9%) [15]. Silva et al. [32] evaluated the EOs of fruits at different ripening stages (unripe and ripe), and identified caryophyllene oxide ranging from 52.1% (unripe) to 27.9% (ripe); β-chenopodiol (17%), which was detected only in unripe fruits; and bicyclogermacrene, which varied from 9.9% (unripe) to 6.9% (ripe).

The chemical distinction of bark EOs was explained by the metabolites V41, V68, V77, V98, V111, V114, V121, and V122, which were exclusive to these samples, along with V18, V20, V22, and V46, which were found at higher concentrations in the bark EOs (Figure 1b; Table 1). In total, 64 volatile compounds were detected in bark EOs. Phenylpropanoids were found only in bark EOs, being the dominant chemical class regardless of season, accounting for 56.7% (Ba-F-1) and 62.0% (Ba-D-1). The most abundant phenylpropanoids were methyl eugenol (V41) (37.5% in Ba-F-1 and 43.6% in Ba-D-1), followed by elemicin (V77) (11.9% in Ba-F-1 and 8.8% in Ba-D-1) and myristicin (V68) (7.4% in Ba-F-1 and 9.6% in Ba-D-1). Sesquiterpene hydrocarbons comprise 15.6% (Ba-F-1) and 12.4% (Ba-D-1), with δ-cadinene (V67) as the main compound with 7.4% in the flooding season and 6.3% in the dry season. Oxygenated sesquiterpenes corresponded to 11.8% (Ba-F-1) and 9.7% (Ba-D-1), while oxygenated monoterpenes showed similar proportions (~11%) in both seasons. Overall, the chemical profiles of the bark EOs were qualitatively consistent between seasons (Table 1, Figure 2).

Essential oils from bark and wood have been the most studied oil from *M. cymbarum* and have presented chemical variations. Zoghbi et al. [12] analyzed bark EO extracted from *M. cymbarum* collected in Pará state, Brazil, and reported a high proportion of sesquiterpene hydrocarbons, such as α-selinene (25.8%) and δ-cadinene (18.6%). On the other hand, Avila et al. [11] identified the monoterpenes α-terpineol (34.9%), α-pinene (18.5%), fenchol (6.3%), and borneol (6.2%) as the major components of wood/bark EOs obtained from plants from Arauca, Colombia. Recently, volatiles extracted by SPME from the sapwood and heartwood of *M. cymbarum* revealed α-copaene (17%), 1,8-cineole (11%), *trans*-calamene (7.4%), α-calacorene (6.4%), and δ-cadinene (5.5%) as the main constituents, each with a relative area greater than 5% [7]. In *M. morae*, the major components of bark EO were the oxygenated monoterpene 1,8-cineole (12.8%) and the hydrocarbon sesquiterpene β-caryophyllene (6.1%) [16]. Bark EO of *M. quixos* was rich in phenylpropanoid derivatives, including *trans*-cinnamaldehyde (44.7%) and *trans*-methyl cinnamate (26.2%) as the dominant constituents [19].

The chemical data were applied to produce molecular networking (Figure S3, Supporting Information), which assisted the visualization of the chemical similarities between the metabolites. Oxygenated monoterpenes formed a distinct group, predominantly marked by metabolites from the leaf EOs, while the phenylpropanoid cluster was clearly separated and composed exclusively of metabolites from the bark EOs. The fruit EOs were more prominently represented in the cluster of hydrocarbon monoterpenes. Oxygenated sesquiterpenes formed another cluster connected to the hydrocarbon sesquiterpenes and included compounds from all EO types.

These inter- and intraspecific chemical variations, including the biosynthesis of secondary metabolites, can be influenced by genotypes and biotic and abiotic factors such as herbivory, pathogens, plant age, phenological stages, temperature, drought, flooding, light exposure, shading, altitude, soil type, salinity, and ultraviolet radiation [22,33]. The biosynthesis of volatile compounds varies by plant organ, with EOs from bark, leaves, and fruits each exhibiting distinct chemical profiles. In general, we did not observe qualitative differences between the EOs from flooding and dry seasons. However, some metabolites showed relative area percentage variation depending on the collection period (Figure 2). For instance, the total content of hydrocarbon sesquiterpenes identified were higher in the EOs of flooding season, whereas hydrocarbon monoterpenes, oxygenated monoterpenes and oxygenated sesquiterpenes were present in greater proportions in the leaf EOs from the dry season (Table 1).

Conversely, the EO yield from the leaves of *Ocotea lancifolia*, collected in the southern region of Brazil, was influenced by seasonality, presenting higher oil content during the spring (1.03%) and summer (0.96%; *w*/*w*%) compared to winter and autumn (0.56% and 0.6%, respectively) [32]. Similarly, the EO yield from young leaves of *Nectandra megapotamica*, also collected in southern Brazil, was higher in spring (0.6%) [34]. Seasonal variations in EO yield were also reported for *Nectandra lanceolata* and *Nectandra grandiflora* collected in southeast Brazil, recording the highest EO yield in samples from spring (0.23% and 0.17%) and autumn (0.2% and 0.17%), respectively [35]. *Nectandra grandiflora* obtained in southern Brazil also exhibited the highest EO content during spring (0.65%) [36]. However, no significant seasonal variation was detected in the oil content of *N. megapotamica* collected in southeast Brazil, demonstrating that seasonal impact can be distinct for each species or population [35].

Although differences in EO yields were observed in several studies, seasonality is not the unique driver of chemical variation, with other factors, such as phenology and pathogen damage, also contributed to differential metabolism [32,36]. Thus, the chemical composition of *O. lancifolia* was not significantly impacted by seasonality, but it was influenced by pathogen attack [32]. Similarly, the major metabolites in the EO of young leaves from *N. megapotamica* from southern Brazil did not show significant differences among seasons; however, some volatiles, including limonene and α- and β-pinene, were higher in spring, and then decreasing until the lowest amount occurred in winter [34]. In contrast, the three *Nectandra* spp. collected in southeastern Brazil exhibited differences in their chemical profiles related to seasonality [35]. For example, no significant differences were observed in the EO yield of *N. megapotamica* across different seasons; however, the chemical composition of the oils varied significantly throughout the year [35].

It is worth noting that in the southern regions of Brazil, where the studies cited above were conducted [32,34,35,36], seasons are markedly distinct, with temperatures and daylight hours varying throughout the year [37]. On the other hand, the Amazon region is near the equator line, and it experiences less pronounced variations in temperature and daylight [37]. Even precipitation is more consistent throughout the year in certain areas, such as the várzea forest, undergoing periodic flooding as a stressor [37]. However, flooding did not appear to affect the oil yield in *M. cymbarum*, possibly because this species may be adapted to grow and develop in high-várzea environments, and it may have mechanisms to tolerant flooding stress. Although no qualitative differences were observed in the volatile profiles between seasons, minor variations in compound percentages can be noted in Figure 2.

### 2.3. Antibiofilm and Antibacterial Activity of Essential Oils from Mespilodaphne cymbarum

We investigated the impact of essential oils (EOs) from leaves, bark, and fruits of *M. cymbarum* on bacterial growth of and biofilm formation by three Gram-positive bacterial strains, *S. epidermidis* (ATCC 35984), *S. aureus* (ATCC 25904), and *M. luteus* (ATCC 4698), and three Gram-negative strains, *E. coli* (ATCC 25922), *P. aeruginosa* (ATCC 27853), and *P. aeruginosa* (PAO1). The strains *S. aureus*, *E. coli*, and *P. aeruginosa* are part of the ESKAPE group of bacteria, which are highly significant in the clinical treatment of infections due to their multiple antibiotic resistance. Therefore, the development of new treatment strategies targeting these pathogens is urgently needed. In contrast, *S. epidermidis* and *M. luteus* are bacterial strains commonly found on human skin and may represent a valuable resource for the discovery of new bioproducts derived from biodiversity. Thus, these bacteria were selected for our study [38,39,40,41].

**Figure 3:**
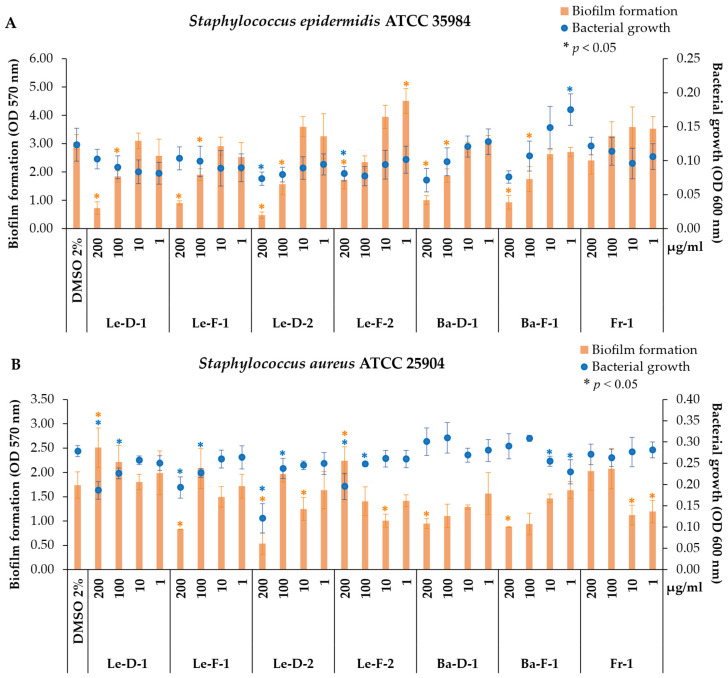

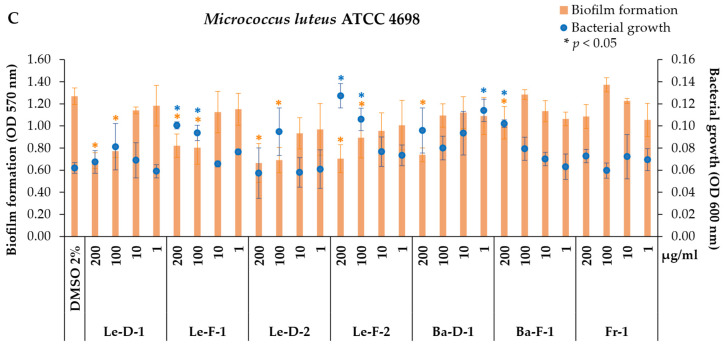
Antibacterial and antibiofilm activities of the essential oils from leaf, bark and fruit of *Mespilodaphne cymbarum* against three Gram-positive bacterial strains: (**A**) *Staphylococcus epidermidis*, (**B**) *Staphylococcus aureus*, and (**C**) *Micrococcus luteus*. Control treatment contains the vehicle dimethyl sulfoxide 2% (DMSO 2%); treatment with the essential oils (EOs): Le-D-1 (leaves from the dry season of chemotype-1), Le-F-1 (leaves from the flooding season of chemotype-1), Le-D-2 (leaves from the dry season of chemotype-2), Le-F-2 (leaves from the flooding season of chemotype-2), Ba-D-1 (bark from the dry season of chemotype-1), Ba-F-1 (bark from the flooding season of chemotype-1), and Fr-1 (fruits from the flooding season of chemotype-1). (*) Significant activity compared with the control (DMSO 2%) by paired *t* tests (* *p* < 0.05). The positive control was vancomycin 8 μg/mL.

In general, the EOs extracted from leaf and bark exhibited higher activity at higher concentrations (200 and 100 μg/mL) compared to fruit EO, demonstrating superior efficacy against Gram-positive than Gram-negative strains (Figure 3; Figure S4). The cell envelope of Gram-positive bacteria lacks an outer membrane and features a thick layer of peptidoglycan along with teichoic acid, which allows the penetration of hydrophobic molecules [38,39]. In contrast, Gram-negative bacteria have a thinner peptidoglycan layer and an outer membrane composed primarily of lipopolysaccharides within a double layer of phospholipids [38,39]. Thus, certain metabolites can inhibit the growth of Gram-positive bacteria but exert a weaker effect on Gram-negative species, particularly due to their hydrophobic properties, enabling penetration within the thick peptidoglycan cell wall of Gram-positive species [40].

In terms of this, the effectiveness of EOs can vary between Gram-positive and Gram-negative bacteria that is commonly linked to the EOs’ volatile composition [40]. For instance, thymol, a volatile compound associated with the antimicrobial properties of several EOs, has demonstrated good effectiveness against Gram-positive bacteria due to its ability to disturb lipids in the plasma membrane [41]. The metabolite β-caryophyllene exhibited strong antibacterial activity, with minimum inhibitory concentrations (MIC) ranging from 3 to 14 μM, showing a more pronounced effect on Gram-positive bacteria [42].

Considered a commensal organism from skin microbiota, *S. epidermidis* is a Gram-positive and coagulase-negative bacterium [43,44]. Under balanced conditions, *S. epidermidis* is often benign, even providing protection against other pathogens, including *S. aureus* [44]. However, *S. epidermidis* can also act as an opportunistic pathogen, frequently related to nosocomial infections due to its ability to produce biofilms, being one of the most common contaminants on medical devices, catheters, and implants [43,44]. In addition, this strain in skin conditions is frequently found in biofilms and is associated with atopic dermatitis [44].

Therefore, samples Le-D-1 and Le-F-1 inhibited biofilm formation of *S. epidermidis*, while the samples Le-D-2 (200 μg/mL; *p* = 0.029) and Le-F-2 (200 μg/mL; *p* = 0.034) affected the bacterial growth by 40% and 34%, respectively. Although Ba-D-1 and Ba-F-1 also reduced bacterial growth by approximately 39% and 37%, these effects were not statistically significant. However, both samples significantly inhibited biofilm formation by approximately 67% (*p* = 0.001) and 70% (*p* = 0.002), respectively. The antibiofilm activity was significative for the highest concentrations tested, with Le-D-1 reducing biofilm formation by 76% (*p* = 0.004) and 39% (*p* = 0.026), and Le-F-1 by 70% (*p* = 0.007) and 37% (*p* = 0.038), at 200 and 100 μg/mL, respectively. Biofilm production is a key virulence factor in *S. epidermidis* and other pathogenic bacteria, protecting this organism from immune defense cells and reducing the action of antibiotics [43]. Since biofilm integrity is crucial for bacterial resistance, compounds capable of disrupting this matrix or inhibiting its formation can be important in the treatment of infections or the prevention of biofilms, and could be applied to biomaterials and medical devices [45].

Considering the major volatile compounds, chemotype-2 exhibited higher percentages of δ-cadinene (V67) and the unknown *m*/*z* 234 (V82), whereas chemotype-1 was characterized by higher levels of α-santalene (V43) and cryptone (V21) (Table 1). Interestingly, although the fruit essential oil displayed the highest percentage of α-santalene (V43), it showed no significant activity against *S. epidermidis*. These results suggest that α-santalene (V43) does not play a critical role in inhibiting biofilm formation in this strain.

Similarly, both chemotypes contain comparable amounts of β-caryophyllene (V42), yet they display distinct biological activities. This suggests that β-caryophyllene may not be solely responsible for the EOs’ effectiveness or could act synergistically with specific compounds unique to each chemotype. In contrast, cryptone (V21) is higher in chemotype-1, while the unknown *m*/*z* 234 (V82) and δ-cadinene (V67) are higher in chemotype-2; thus, they could be related to the distinct activity of each oil. A moderate activity against *S. aureus* (ATCC 25932) for essential oil from *Eucalyptus odorata*, which showed around 20% cryptone, was also described [46]. Another possibility is the synergistic effect of metabolites since we found a notable difference between chemotype-1 and -2, as well as some volatiles exclusive to each chemotype (Table 1, Figure 2).

In contrast, bark EOs presented methyl eugenol (V41) (approx. 40%), followed by elemicin (V77) (Ba-F-1 = 12.1%; Ba-D-1 = 9%), myristicin (V68) (Ba-F-1 = 7.5%; Ba-D-1 = 10.5%), and δ-cadinene (V67) (Ba-F-1 = 7.6%; Ba-D-1 = 6%). The EO from *Malaleuca bracteata* showed approximately 88% methyl eugenol and demonstrated moderate activity against *S. epidermidis* with a MIC value of 500 μg/mL [47]. Interestingly, the authors of the same study also evaluated the pure methyl eugenol and its MIC against *S. epidermidis* was higher than that of the EO (1000 μg/mL), suggesting a possible synergism among the volatiles present in this EO [47]. Another species with chemotypes rich in methyl eugenol (≈39%) is *Ocimum basilicum*, which demonstrated bactericidal activity against *S. epidermidis* with a minimum bactericidal concentration (MBC) of approximately 416 μg/mL [48]. Additionally, this EO showed better results against Gram-positive bacteria compared to Gram-negative bacteria [48].

In the assays against *S. aureus* (ATCC 25904), all leaf EOs at higher concentrations (200 and 100 μg/mL) significantly decreased bacterial growth to some extent: Le-D-1 (33%, *p* = 0.015; and 19%, *p* = 0.033), Le-F-1 (30%, *p* = 0.040; and 18%, *p* = 0.049), Le-D-2 (57%, *p* = 0.014; and 15%, *p* = 0.023), and Le-F-2 (30%, *p* = 0.044; and 11%, *p* = 0.019) (Figure 3B). Leaf EOs of *M. cymbarum* from both chemotypes reduced *S. aureus* growth to different extents. Among the major metabolites in these EOs, we identified β-caryophyllene, which is known for its biological properties, such as anti-inflammatory, antioxidant, cytotoxic, antifungal, and antimicrobial activities [42,49,50]. The structure of β-caryophyllene facilitates the penetration of cell membranes, potentiating the effects of other drugs [49]. Other essential oils containing high levels of β-caryophyllene, as well as this isolated metabolite, exhibited varying degrees of activity, being described as potent against *S. aureus* (MTCC 7405) with a MIC of 3 μM [42], but weak activity against *S. aureus* (ATCC 25923) with a MIC of 1 mg/mL [50]. Moreover, the EO of *Murraya paniculata* showed higher biological activity compared to the isolated β-caryophyllene, indicating a synergistic effect of its chemical components [50].

Additionally, antibiofilm activity was observed at the highest concentrations of bark EOs, with reductions of 45% (Ba-D-1; *p* = 0.049) and 49% (Ba-F-1; *p* = 0.032), and at the lowest concentrations of fruit EO, decreasing biofilm formation by 35% at 10 μg/mL (*p* = 0.014) and 31% at 1 μg/mL (*p* = 0.032) (Figure 3B). Bark EOs, rich in phenylpropanoids, particularly methyl eugenol (V41), demonstrated typical antibiofilm activity by reducing biofilm formation without inhibiting bacterial growth. EO from *L. nobilis* from plants collected in Sousse (Tunisia) presented as major volatiles 1,8-cineole (30.8%), methyl eugenol (15.6%), and α-terpinyl acetate (14.5%), and evidenced low antibacterial activity (MIC = 31.25 mg/mL), and moderate biofilm inhibition up to 70% at a concentration of 1.95 mg/mL [51].

Another Gram-positive strain evaluated in our study was *M. luteus*, a bacterium of the mammalian skin microbiome that is an obligate aerobic species, nonmotile, non-spore-forming, and catalase and oxidase-positive [52]. Generally, *M. luteus* is not considered harmful to humans, but it can act as an opportunistic pathogen in certain cases, particularly in immunocompromised individuals, causing pneumonia, bacteremia, endocarditis, peritonitis, ventriculitis, and septic arthritis [53]. This species is capable of producing biofilms and colonizing, for instance, prosthetic material, leading to infections such as endocarditis [53].

The EOs of *M. cymbarum* presented antibiofilm activity against *M. luteus* (ATCC 4698), reducing biofilm formation. No significant activity against *M. luteus* was observed for Fr-1, but all leaf EOs demonstrated antibiofilm activity at higher concentrations (Figure 3C), diminishing the biofilm formation rate by 30% to 47% (*p*-values ranging from 0.009 to 0.04). Additionally, Ba-D-1 and Ba-F-1 at 200 μg/mL reduced biofilm formation by 42% (*p* = 0.001) and 19% (*p* = 0.031), respectively. This antibiofilm potential could be explored as a strategy against this pathogen to prevent its formation of biofilms, such as by application to biomaterials [40].

In contrast, the EOs of *M. cymbarum* were not active against the Gram-negative bacterial strains, except for *P. aeruginosa* (ATCC 27853) (Figure S4; Supplementary Information). The leaf EOs from the dry season (Le-D-1 and Le-D-2) at 200 μg/mL reduced *P. aeruginosa* (ATCC 27853) growth by 42% and 43%, respectively (Figure S4A). Biofilm formation was inhibited by Ba-F-1 by approximately 19% at 10 μg/mL and by Fr-1 by about 22% at 100 μg/mL and 20% at 10 μg/mL (Figure S4A). The EOs from *M. cymbarum* did not decrease the bacterial growth of *P. aeruginosa* (PAO1) and *E. coli* (ATCC 25922), and even stimulated biofilm formation at some concentrations (Figure S4B,C). Several studies have reported that EOs tend to be less effective against Gram-negative than Gram-positive bacteria, presumably due to the differences in their membrane composition [40,48].

## 3. Materials and Methods

### 3.1. Study Area

The samplings of *M. cymbarum* were carried out in the Mamirauá Sustainable Development Reserve, which covers approximately 1,124,000 hectares in the Central Amazon, at the confluence of the Solimões, Japurá, and Auati-Paraná rivers [54]. The climate is classified as tropical humid, with annual average temperatures ranging from 28 to 30 °C. Rainfall occurs throughout the year, but it is more abundant during specific periods, resulting in a monomodal flood pulse. Throughout the year in this Amazon region, a rising water period typically begins in January and continues until May, when the peak of flooding occurs and remains until July. Between July and September, the water level begins to recede, marking the low water period, with the peak of the dry season occurring between September and November. It is important to note that these seasonal fluctuations may vary slightly from year to year, primarily due to differences in rainfall [54].

### 3.2. Processing the Vegetal Material

We collected leaves and bark from 14 individuals with different diameters at breast height (DBH) to minimize the influence of plant age and ensure the inclusion of samples from different developmental stages (Table S1, Supplementary Materials). The access to Brazilian genetic heritage was registered in the National Management System for Genetic Heritage and Associated Traditional Knowledge (SisGen) under the number A8B202A. Both leaves and bark were collected during the dry (September 2021) and flooding (April 2022) seasons. Fruits were only available during the flooding season (April 2022) and we successfully collected fruits from eight specimens of chemotype-1 (*n* = 8). The plant materials (bark and leaves) were dried in a controlled environment at 24 °C. Due to their fleshy nature, we subjected the fruits to drying in an air-circulating oven at 45 °C until they reached a consistent weight. Then, the dried materials were powdered by a knife mill. Additionally, fertile specimens were deposited in the Herbarium of Campo Grande, MS, under the catalog number (CGMS–83228). The plant identification was confirmed by Professor Flávio Macedo Alves, a specialist in Lauraceae.

### 3.3. Extraction and Yield of Essential Oils (EOs) from Mespilodaphne cymbarum

The EOs were extracted by hydrodistillation for 4 h using a Clevenger apparatus. Leaves from different individuals were grouped based on the two chemotypes identified from a pilot study (data shown on Figure S1). Then, the samples were extracted separately, resulting in four distinct sample groups: leaves from the dry season of chemotype-1 (Le-D-1) and chemotype-2 (Le-D-2); and leaves from the flood season of chemotype-1 (Le-F-1) and chemotype-2 (Le-F-2). The fruits were available only during the flood season and from chemotype-1 (Fr-1). Bark EOs were exclusively from chemotype-1, collected in both the dry (Ba-D-1) and flood (Ba-F-1) seasons, as insufficient bark material was available for chemotype-2. The EOs were filtered through anhydrous sodium sulfate to remove water residue and stored in amber glass containers at −18 °C until analysis. The extracted oil volume was used to calculate the yield as a percentage relative to the dry plant weight (% *v*/*w*).

### 3.4. Analysis of the Essential Oils (EOs) by Gas Chromatography Coupled to Mass Spectrometry (GC-MS)

The EOs were analyzed using a Shimadzu model QP2010 (Shimadzu, Tokyo, Japan) gas chromatograph coupled to a mass spectrometer (GC-MS), which was equipped with an RTx-5MS chromatographic column (30 m × 0.25 mm i.d., 0.25 μm film thickness) and an ionization energy of 70 eV. The temperature program was initiated at 60 °C, increasing at a rate of 3 °C/min up to 240 °C. The injector and interface temperatures were set at 250 °C. Helium was used as carrier gas with a linear velocity of 41.6 cm/s and a pressure of 79.7 K Pa. Each EO was prepared at 10 mg/mL and 1 μL was injected into the GC-MS, applying a split ratio of 1:10. The samples were injected in duplicates and all the samples were used to prepare a quality control sample, which was injected during the analysis. A series of C8–C40 n-alkane standards were injected and used to calculate the retention indices.

The compounds were annotated based on the comparison of mass spectra deposited in the NIST08, FFNSC 1.3, and WILEY 7 libraries, and of their retention indices [55,56].

### 3.5. Data Processing

We aligned the GC-MS data using MetAlign 3.0 software and reduced the entrances with MSClust, resulting in 349 entrances. Then, we removed the duplicate entrances, those with a low probability of being identified as compounds (cent.factor < 0.9), and peaks with ion intensity lower than 10,000. Next, we crossed this data with the chromatographic data processed in software GC Solution Version 4.20 (Shimadzu), in which peak areas were integrated and only peaks with a relative area greater than 0.3% were selected, resulting in a total of 122 features. For peak integration, we applied a slope parameter based on the sample type: for leaves, the slope was set to max height/1000, and for bark and fruits, it was set to max height/2000. For statistical analysis, we excluded all peaks with a relative area below 0.3%.

### 3.6. Molecular Networking

The GC-MS data of the EOs from bark, leaves, and fruits were used to create a molecular network at the Global Natural Products Social Molecular Networking (GNPS) platform (https://gnps.ucsd.edu, accessed on 23 April 2025) [57]. The MS/MS fragment ions within +/− 17 Da of the precursor *m*/*z* were removed. Additionally, the MS/MS spectra were refined by retaining only the top six fragment ions in the +/− 50 Da window throughout the spectrum, and the MS/MS fragment ion tolerance was 1 Da. A molecular network was built by retaining only edges with a cosine score greater than 0.7 and more than six matched peaks. Moreover, edges between two nodes were included only if each node was among the other’s top 10 most similar nodes. The maximum size of each molecular family was limited to 100 nodes. The library spectra were filtered using the same method as the input data. Only the matches with a score above 0.5 and at least six matched peaks were kept to the network spectra and library spectra. The network and library spectra results are available at the following link https://gnps.ucsd.edu/ProteoSAFe/status.jsp?task=3892fb335b6d47b2a2f76a0631165574 (accessed on 23 April 2025). The molecular network was visualized and edited using Cytoscape software, version 3.10.2 [58].

### 3.7. Bacterial Strains

Six bacterial strains were used, including three Gram-positive—*Staphylococcus epidermidis* (ATCC 35984), *Staphylococcus aureus* (ATCC 25904), and *Micrococcus luteus* (ATCC 4698); and three Gram-negative—*Escherichia coli* (ATCC 25922), *Pseudomonas aeruginosa* (ATCC 27853), and *Pseudomonas aeruginosa* (PAO1).

### 3.8. Bacterial Growth and Biofilm Formation Assays

The evaluation of EOs on bacterial growth and biofilm formation was carried out using crystal violet microplate assays, adapted from Trentin et al. [59]. Crystal violet (Newprov, 0.4% *w*/*v*) was freshly prepared by diluting the stock solution in distilled water. The EOs were initially diluted in 100% dimethyl sulfoxide (DMSO) and afterwards diluted to a final concentration of 2% DMSO for use in the assays. These solutions were then diluted to achieve final concentrations of 200, 100, 10 and 1 μg/mL, and 4 μL of each sample was placed in a 96-well microplate. Wells containing 4 μL of 2% DMSO were used as evidence for growth control. The antibiotics vancomycin and meropenem (8 μg/mL) were used as positive controls against Gram-positive and Gram-negative bacteria, respectively. Subsequently, 80 μL of sterile water and 40 μL of tryptone soy broth (TSB) culture medium (Oxoid Ltd., England) were added, except for the experiment with *S. aureus* ATCC 25904 that was carried out with Brain Heart Infusion Broth culture medium (BHI) supplemented with 1% glucose. Then, a bacterial suspension with an absorbance of 0.15 ± 0.01 (OD 600 nm) was added and the final absorption was read. The plates were incubated at 37 °C for 24 h.

After 24 h, the absorbance (OD 600 nm) was taken to evaluate bacterial growth. Subsequently, the contents of the wells were removed and then the wells were washed three times with saline and incubated for 1 h to dry the adhered biofilm in a 60 °C oven. Following the addition of 200 μL of crystal violet, the samples were incubated for 15 min. The excess stain was removed through vigorous washing, and 200 μL of absolute ethanol was then added to solubilize the retained dye. Therefore, a new absorbance reading (OD 570 nm) was taken to quantify the violet crystal adhered to the biofilm. The results were compared with the control for the presence of 2% DMSO. Inhibition percentages for both bacterial growth and biofilm formation were calculated using the following formula:

% inhibition = [(OD control − OD sample)/OD control] × 100.

### 3.9. Statistical Analysis

The peak intensity matrix was statistically analyzed using R software version 4.2.3 (https://www.r-project.org/, accessed on 10 September 2024) [60]. The data were log-transformed (base 10) and scaled to minimize the influence of extreme values. Principal Component Analysis (PCA) was performed on the volatile intensities of the essential oils (EOs) extracted from leaves, bark, and fruits, using the “stats” [60] and “factoextra” packages [61]. To assess differences among EOs from bark, leaves, and fruits, a Permutational Analysis of Variance (PERMANOVA) was conducted using the “vegan” package [62]. The analysis was based on Bray–Curtis dissimilarities, with 999 permutations and a significance level set at *p* < 0.05.

Hierarchical Clustering Analysis (HCA), along with a heatmap, was carried out in MetaboAnalyst 6.0 [63] using the matrix of relative area (%) for each volatile compound. Initially, we analyzed the EOs from bark, fruits, and leaves. Then, we conducted HCA and generated a heatmap for the leaf EOs from both chemotypes to illustrate the distinctions among these groups, exhibited in the supplementary information.

The potential antimicrobial and antibiofilm activities were evaluated using a paired *t* test to compare each sample and concentration against the control (containing 2% DMSO). Absorbance values were used, and a significance threshold of *p* < 0.05 was applied. These analyses were conducted in Excel.

## 4. Conclusions

Our results elucidated, for the first time, the metabolite profiles of the leaf and fruit EOs of *M. cymbarum*, revealing significant distinctions between plant organs and the presence of two distinct chemotypes. These findings reinforce the importance of analyzing different plant tissues to capture chemical variability. The distinct chemical compositions of EOs extracted from leaf, bark, and fruit of *M. cymbarum* resulted in different levels of activity against the evaluated pathogenic bacterial strains. Although the fruit EO demonstrated the weakest activity against the tested bacterial strains, it yielded a high oil content and featured an interesting chemical profile, including considerable amounts of valuable sesquiterpenes, such as α- and β-santalene, α-*trans*-bergamotene, and α-santalol, which are widely used in the perfume and cosmetics industries. The leaf and bark EOs showed higher activity, particularly against Gram-positive bacteria, acting either on bacterial growth and/or biofilm formation. Notably, the biological activity varied between the two leaf EO chemotypes, likely reflecting their chemical divergence. While major constituents are often associated with antimicrobial activity, it is plausible that minor compounds contribute synergistically to the overall effect. The enhanced activity observed in leaf EOs may also be linked to their greater chemical complexity, as they contained a higher number of volatile constituents compared to bark and fruit EOs. Our findings open new possibilities for exploring the leaves of *M. cymbarum* as a sustainable source of EOs. Even the fruits could be used through proper management, since only the pulp was analyzed in this study, thus allowing the preservation of the seeds. In this context, future studies could propose sustainable strategies for the use of this species, offering alternative ways to exploit its EOs that could benefit Amazonian communities. Moreover, further investigations are warranted to assess toxicity, determine the minimum inhibitory concentration (MIC), and develop biomaterials capable of preventing bacterial biofilm formation or strategies to combat pathogenic bacterial growth using these essential oils. These findings not only highlight the potential of *M. cymbarum* EOs but also pave the way for future research and innovation in this field.

## Figures and Tables

**Table 1:** Chemical composition and yield (%) of the essential oils (EOs) extracted from leaves (flooding and dry), bark (flooding and dry), and fruits of *Mespilodaphne cymbarum*, collected in the Mamirauá Sustainable Development Reserve, Uarini, AM, Brazil. These EOs were analyzed by GC-MS using a RTx-5MS chromatographic column.

VOC	IR *	Compound Name	Le-F-1	Le-D-1	Le-F-2	Le-D-2	Fr-1	Ba-F-1	Ba-D-1	Class **
V1	938	α-Pinene	1.71 ± 0.021	1.88 ± 0.247	0.1 ± 0.007	0.38 ± 0.007	3.32 ± 0.583	0.21 ± 0.021	0.08 ± 0.001	HM
V2	979	β-Pinene	0.83 ± 0.007	0.87 ± 0.078	<0.1	0.26 ± 0.007	1.2 ± 0.289	0.13 ± 0.007	0.08 ± 0.007	HM
V3	990	Myrcene	-	-	-	-	0.6 ± 0.134	<0.1	<0.1	HM
V4	1004	α-Phellandrene	0.11 ± 0.007	0.11 ± 0.007	<0.1	<0.1	1.76 ± 0.246	-	-	HM
V5	1026	*p*-Cymene	2.64 ± 0.078	2.59 ± 0.198	0.2 ± 0.007	2.04 ± 0.042	0.96 ± 0.237	<0.1	<0.1	HM
**V6**	**1031**	**Limonene**	2.37 ± 0.042	2.89 ± 0.233	0.33 ± 0.007	1.38 ± 0.028	**19.32 ± 2.79**	0.17 ± 0	0.22 ± 0.014	**HM**
V7	1034	1,8-Cineole	1.73 ± 0.007	1.9 ± 0.106	0.22 ± 0.007	0.76 ± 0.021	0.62 ± 0.114	3.33 ± 0.177	3.44 ± 0.184	OM
V8	1050	*trans*-β-Ocimene	-	-	-	-	0.54 ± 0.179	-	-	HM
V9	1074	*cis*-Linalool oxide	0.38 ± 0.007	0.28 ± 0.007	0.21 ± 0.002	0.21 ± 0.007	-	-	-	OM
V10	1088	*trans*-Linalool oxide	0.27 ± 0.007	0.22 ± 0.001	0.17 ± 0.001	0.16 ± 0.001	-	-	-	OM
V11	1088	Terpinolene	-	-	-	-	0.5 ± 0.074	-	-	HM
V12	1098	Linalool	0.11 ± 0.007	0.34 ± 0.007	<0.1	0.1 ± 0.007	<0.1	0.1 ± 0.007	0.08 ± 0.014	OM
V13	1141	*trans*-Pinocarveol	0.86 ± 0.021	1.13 ± 0.113	<0.1	<0.1	<0.1	<0.1	<0.1	OM
V14	1144	*cis*-Verbenol	0.26 ± 0.007	0.37 ± 0.001	<0.1	<0.1	-	-	-	OM
V15	1148	*trans*-Verbenol	0.63 ± 0.014	1.3 ± 0.014	<0.1	0.2 ± 0.007	-	-	-	OM
V16	1153	NI (*m*/*z* 138)	0.67 ± 0.035	0.8 ± 0.007	<0.1	<0.1	<0.1	-	-	
V17	1165	Pinocarvone	0.27 ± 0.007	0.49 ± 0.001	<0.1	<0.1	-	-	-	OM
V18	1169	Borneol	<0.1	<0.1	<0.1	<0.1	<0.1	0.88 ± 0.049	0.65 ± 0.396	OM
V19	1170	*p*-Mentha-1,5-dien-8-ol	0.55 ± 0.021	0.5 ± 0.007	<0.1	<0.1	-	<0.1	0.35 ± 0.042	OM
V20	1179	Terpinen-4-ol	0.55 ± 0.007	0.76 ± 0.007	0.18 ± 0.007	0.41 ± 0.021	0.18 ± 0.031	2.07 ± 0.014	1.97 ± 0.021	OM
**V21**	**1188**	**Cryptone**	**5.03 ± 0.014**	**5.08 ± 0.134**	1.94 ± 0.028	2.64 ± 0.035	<0.1	<0.1	<0.1	Ke
V22	1192	α-Terpineol	0.42 ± 0.014	0.64 ± 0.007	0.23 ± 0.007	0.29 ± 0.007	0.75 ± 0.049	4.5 ± 0.078	4.38 ± 0.042	OM
V23	1197	Myrtenal	0.72 ± 0.014	1.1 ± 0.007	<0.1	<0.1	<0.1	<0.1	<0.1	OM
V24	1210	Verbenone	1.29 ± 0.021	0.86 ± 0.007	<0.1	<0.1	-	-	-	OM
V25	1221	*trans*-Carveol	0.62 ± 0.007	0.7 ± 0.007	0.22 ± 0.007	0.34 ± 0.014	<0.1	-	-	OM
V26	1226	NI	0.57 ± 0.007	0.56 ± 0.007	-	-	-	-	-	
V27	1243	*p*-Isopropylbenzaldehyde (*p*-Cumic aldehyde)	1.94 ± 0.057	2.58 ± 0.028	0.95 ± 0.035	1.62 ± 0.049	-	-	-	OM
V28	1247	Carvone	0.8 ± 0.028	0.89 ± 0.007	0.35 ± 0.007	0.52 ± 0.014	<0.1	-	-	OM
V29	1276	*p*-Menth-1-en-7-al	0.63 ± 0.007	0.67 ± 0.001	<0.1	0.26 ± 0.014	<0.1	-	-	OM
V30	1292	*p*-Cymen-7-ol	0.61 ± 0.028	0.64 ± 0.014	0.32 ± 0.007	0.52 ± 0.035	-	-	-	OM
V31	1308	NI	0.74 ± 0.014	0.59 ± 0.007	-	-	-	-	-	
V32	1331	NI	1.04 ± 0.014	1.39 ± 0.014	<0.1	<0.1	<0.1	-	-	
V33	1334	NI	0.61 ± 0.007	1.05 ± 0.028	<0.1	<0.1	<0.1	-	-	
V34	1337	δ-Elemene	-	-	0.26 ± 0.007	0.26 ± 0.014	0.37 ± 0.045	-	-	HS
V35	1339	NI	1.57 ± 0.007	2.12 ± 0.014	-	-	-	-	-	
V36	1351	α-Cubebene	0.18 ± 0.007	0.21 ± 0.014	<0.1	<0.1	-	1.1 ± 0.049	1.12 ± 0.014	HS
V37	1368	Cyclosativene	0.32 ± 0.007	0.33 ± 0.007	0.62 ± 0.007	0.68 ± 0.007	-	<0.1	<0.1	HS
V38	1370	NI	1.07 ± 0.035	0.35 ± 0.001	-	-	<0.1	-	-	
V39	1376	α-Copaene	1.39 ± 0.021	0.91 ± 0.014	1.65 ± 0.021	1.7 ± 0.014	0.26 ± 0.024	0.95 ± 0.049	0.53 ± 0.014	HS
V40	1391	β-Elemene	0.18 ± 0.007	<0.1	0.22 ± 0.007	<0.1	0.32 ± 0.051	<0.1	<0.1	HS
**V41**	**1411**	**Methyl eugenol**	-	-	-	-	-	**37.49 ± 1.29**	**43.61 ± 0.49**	**Ph**
**V42**	**1419**	**β-Caryophyllene**	**7.91 ± 0.926**	**4.77 ± 0.106**	**8.99 ± 0.071**	**7.61 ± 0.113**	2.97 ± 0.093	-	-	**HS**
**V43**	**1421**	**α-Santalene**	**6.17 ± 0.085**	**6.29 ± 0.226**	<0.1	<0.1	**26.41 ± 0.50**	1.69 ± 0.028	1.11 ± 0.028	**HS**
**V44**	**1437**	***trans*** **-α-Bergamotene**	2.85 ± 0.042	1.94 ± 0.014	0.79 ± 0.007	0.33 ± 0.007	**8.18 ± 0.181**	0.27 ± 0.021	0.2 ± 0.014	**HS**
V45	1439	α-Guaiene	0.68 ± 0.007	0.45 ± 0.001	0.21 ± 0.001	<0.1	1.95 ± 0.054	<0.1	<0.1	HS
V46	1444	6,9-Guaiadiene	0.29 ± 0.007	0.15 ± 0.021	0.16 ± 0.001	<0.1	<0.1	0.79 ± 0.042	0.79 ± 0.014	HS
V47	1448	epi-β-Santalene	0.3 ± 0.007	0.37 ± 0.049	<0.1	<0.1	1.61 ± 0.107	-	-	HS
V48	1451	Allo-Aromadendrene	<0.1	0.3 ± 0.035	0.31 ± 0.007	0.28 ± 0.021	-	-	-	HS
V49	1454	α-Humulene	0.72 ± 0.007	0.45 ± 0.021	0.92 ± 0.007	0.85 ± 0.021	<0.1	0.16 ± 0.007	<0.1	HS
V50	1458	*trans*-β-Farnesene	0.24 ± 0.007	0.14 ± 0.021	0.35 ± 0.007	0.22 ± 0.001	1.47 ± 0.115	-	-	HS
**V51**	**1462**	**β-Santalene**	3.77 ± 0.014	2.74 ± 0.021	2.05 ± 0.007	1.24 ± 0.014	**12.02 ± 0.64**	0.78 ± 0.049	0.45 ± 0.021	**HS**
V52	1470	Naphthalene, 1,2,3,4,6,7,8,8a-octahydro-1,8a-dimethyl-7-(1-methylethenyl)-, (1R,7S,8aS)-	0.58 ± 0.007	0.9 ± 0.001	2.89 ± 0.014	3.73 ± 0.085	<0.1	<0.1	<0.1	HS
V53	1477	γ-Muurolene	1.45 ± 0.014	0.71 ± 0.007	2.19 ± 0.007	1.75 ± 0.071	0.65 ± 0.092	0.25 ± 0.007	<0.1	HS
V54	1480	α-Amorphene	0.31 ± 0.021	<0.1	0.54 ± 0.007	0.36 ± 0.007	0.14 ± 0.158	<0.1	0.07 ± 0	HS
V55	1485	β-Selinene	2.85 ± 0.021	3.14 ± 0.007	1.49 ± 0.021	1.34 ± 0.021	0.84 ± 0.241	0.06 ± 0.014	0.05 ± 0	HS
V56	1488	Eremophilene	0.3 ± 0.007	0.27 ± 0.007	<0.1	<0.1	0.66 ± 0.18	-	-	HS
V57	1491	β-cadinene	0.45 ± 0.014	0.27 ± 0.001	0.44 ± 0.007	<0.1	0.42 ± 0.132	0.2 ± 0.014	0.16 ± 0.014	HS
V58	1494	Viridiflorene	1.09 ± 0.007	0.73 ± 0.014	1.3 ± 0.014	0.67 ± 0.014	1 ± 0.202	0.19 ± 0.035	0.17 ± 0.007	HS
V59	1498	NI	0.64 ± 0.021	0.58 ± 0.028	0.9 ± 0.021	0.74 ± 0.007	-	-	-	
V60	1498	α-Selinene	-	-	-	-	0.38 ± 0.065	-	-	HS
V61	1499	α-Muurolene	0.51 ± 0.007	0.23 ± 0.001	0.78 ± 0.049	0.56 ± 0.014	<0.1	0.17 ± 0.007	0.16 ± 0.007	HS
V62	1505	α-Bulnesene	<0.1	<0.1	<0.1	<0.1	0.39 ± 0.056	-	-	HS
V63	1509	β-Bisabolene	0.43 ± 0.014	0.26 ± 0.021	0.66 ± 0.007	0.26 ± 0.001	2.12 ± 0.221	<0.1	0.01 ± 0	HS
V64	1514	γ-Cadinene	1.28 ± 0.028	0.88 ± 0.014	3.1 ± 0.014	2.93 ± 0.007	<0.1	0.34 ± 0.007	0.21 ± 0.007	HS
V65	1517	NI	0.63 ± 0.007	0.93 ± 0.014	0.32 ± 0.007	0.51 ± 0.014	<0.1	-	-	
V66	1521	NI	2.61 ± 0.007	3.02 ± 0.035	-	-	-	-	-	
**V67**	**1525**	**δ-Cadinene**	3.37 ± 0.049	1.93 ± 0.021	**5.14 ± 0.014**	**3.62 ± 0.028**	0.52 ± 0.119	**7.41 ± 0.332**	**6.29 ± 0.304**	**HS**
**V68**	**1528**	**Myristicin**	-	-	-	-	-	**7.37 ± 0.205**	**9.6 ± 0.219**	**Ph**
V69	1534	*trans*-γ-Bisabolene	0.98 ± 0.014	0.37 ± 0.007	2.61 ± 0.028	1.21 ± 0.021	1.97 ± 0.234	0.43 ± 0.028	0.21 ± 0.014	HS
V70	1540	α-Cadinene	0.27 ± 0.021	0.22 ± 0.014	0.45 ± 0.021	0.38 ± 0.021	<0.1	-	-	HS
V71	1543	NI	1.26 ± 0.007	1.46 ± 0.113	2.06 ± 0.021	2.14 ± 0.007	<0.1	0.32 ± 0.064	0.19 ± 0	
V72	1545	α-Calacorene	0.49 ± 0.028	0.75 ± 0.057	0.79 ± 0.007	0.87 ± 0.078	-	0.3 ± 0.042	0.16 ± 0.007	HS
V73	1545	*trans*-α-bisabolene	-	-	-	-	0.42 ± 0.113	-	-	HS
V74	1547	NI	-	-	2.99 ± 0.014	3.08 ± 0.035	-	-	-	
V75	1551	α-Elemol	<0.1	<0.1	<0.1	<0.1	0.54 ± 0.318	0.7 ± 0.042	0.79 ± 0.028	OS
V76	1558	NI	3.35 ± 0.007	3.36 ± 0.071	3.32 ± 0.028	3.45 ± 0.007	-	0.5 ± 0.028	0.54 ± 0.014	
**V77**	**1564**	**Elemicin**	-	-	-	-	-	**11.89 ± 0.17**	**8.82 ± 0.014**	**Ph**
V78	1565	NI	1.53 ± 0.028	1.47 ± 0.057	-	-	-	-	-	
V79	1567	NI	0.83 ± 0.014	0.8 ± 0.007	-	-	<0.1	-	-	
V80	1579	Spathulenol	0.19 ± 0.014	0.3 ± 0.028	0.83 ± 0.014	0.66 ± 0.014	<0.1	<0.1	<0.1	OS
V81	1584	Caryophyllene oxide	1.39 ± 0.205	2.92 ± 0.113	2.88 ± 0.007	3.96 ± 0.389	-	<0.1	0.22 ± 0.007	OS
**V82**	**1589**	**NI (*m*/*z* = 234)**	**5.38 ± 0.191**	**5.87 ± 0.134**	**10.99 ± 0.057**	**9.84 ± 0.134**	-	0.47 ± 0.042	0.58 ± 0.007	
V83	1603	Rosifoliol	0.26 ± 0.021	0.3 ± 0.007	1.41 ± 0.071	1.11 ± 0.049	-	0.87 ± 0.049	0.91 ± 0.049	OS
V84	1605	NI	-	-	1.04 ± 0.007	0.83 ± 0.099	-	0.45 ± 0.035	0.24 ± 0.021	
V85	1605	NI	0.85 ± 0.021	0.86 ± 0.035	-	-	-	-	-	
V86	1608	NI	-	-	0.54 ± 0.014	0.74 ± 0.078	-	-	-	
V87	1611	Humulene epoxide II	<0.1	<0.1	0.46 ± 0.021	0.79 ± 0.035	-	0.29 ± 0.007	0.2 ± 0.028	OS
V88	1614	Tetradecanal	-	-	-	-	0.21 ± 0.065	-	-	
V89	1614	Viridiflorol	0.53 ± 0.028	0.49 ± 0.035	0.47 ± 0.002	0.36 ± 0.028	-	-	-	OS
V90	1618	1,10-di-epi-Cubenol	-	-	1.29 ± 0.156	1.19 ± 0.064	-	0.81 ± 0.042	0.73 ± 0.134	OS
V91	1620	NI	-	-	1.84 ± 0.17	2.06 ± 0.042	-	-	-	
V92	1622	epi-γ-Eudesmol	-	-	0.77 ± 0.014	0.68 ± 0.035	-	-	-	OS
V93	1623	NI (*m*/*z* = 236)	<0.1	<0.1	1.04 ± 0.014	0.96 ± 0.021	-	-	-	
V94	1630	1-epi-Cubenol	<0.1	<0.1	0.52 ± 0.007	0.39 ± 0.007	0.2 ± 0.082	0.84 ± 0.156	0.48 ± 0.021	OS
V95	1634	γ-Eudesmol	-	-	-	-	0.5 ± 0.258	0.23 ± 0.064	0.1 ± 0.014	OS
V96	1638	Hinesol	-	-	0.65 ± 0.021	1.17 ± 0.035	-	-	-	OS
V97	1643	1,2,3,4,4a,7,8,8a-octahydro-1,6-dimethyl-4-(1-methylethyl)-1-Naphthalenol,	-	-	0.33 ± 0.007	0.18 ± 0.014	-	<0.1	<0.1	OS
V98	1645	epi-α-Cadinol	-	-	-	-	-	1.19 ± 0.064	0.61 ± 0.014	OS
V99	1645	NI	-	-	0.59 ± 0.028	0.7 ± 0.028	-	-	-	
V100	1649	α-Muurolol (=Torreyol)	<0.1	<0.1	<0.1	<0.1	-	0.33 ± 0.057	0.17 ± 0.014	OS
V101	1651	NI	-	-	3.01 ± 0.042	2.87 ± 0.001	-	-	-	
V102	1652	β-Eudesmol	-	-	2.43 ± 0.057	2.62 ± 0.092	0.62 ± 0.398	2.27 ± 0.12	2.52 ± 0.028	OS
V103	1655	α-Eudesmol	-	-	0.5 ± 0.071	0.73 ± 0.021	0.62 ± 0.419	<0.1	<0.1	OS
V104	1657	α-Cadinol	2.74 ± 0.028	2.9 ± 0.092	2.07 ± 0.049	1.8 ± 0.028	<0.1	1.53 ± 0.085	0.73 ± 0.028	OS
V105	1660	NI	<0.1	<0.1	1.28 ± 0.028	1.58 ± 0.021	-	<0.1	<0.1	
V106	1664	NI	<0.1	<0.1	0.4 ± 0.057	0.56 ± 0.035	-	-	-	
V107	1671	NI	-	-	0.49 ± 0.014	0.27 ± 0.049	-	-	-	
V108	1671	β-Bisabolol	0.46 ± 0.042	0.51 ± 0.014	-	-	0.47 ± 0.242	<0.1	0.07 ± 0.007	OS
V109	1676	Cadalene	0.19 ± 0.078	0.13 ± 0.028	0.91 ± 0.014	0.7 ± 0.007	-	0.17 ± 0.035	0.08 ± 0.007	HS
V110	1678	*cis*-α-Santalol	-	-	-	-	0.78 ± 0.161	-	-	OS
V111	1678	Bulnesol	-	-	-	-	-	1.69 ± 0.247	1.47 ± 0.099	OS
V112	1680	NI	1.04 ± 0.099	1.41 ± 0.014	2.08 ± 0.007	1.64 ± 0.035	-	-	-	
V113	1680	NI	-	-	-	-	0.6 ± 0.171	-	-	
V114	1680	NI	-	-	-	-	-	0.65 ± 0.085	0.6 ± 0.163	
V115	1687	α-Bisabolol	-	-	0.61 ± 0.014	0.55 ± 0.014	<0.1	-	-	OS
V116	1708	NI	-	-	0.76 ± 0.001	0.87 ± 0.014	-	-	-	
V117	1710	NI	0.85 ± 0.014	1.12 ± 0.057	-	-	-	-	-	
V118	1729	NI	-	-	0.62 ± 0.014	0.62 ± 0.014	-	-	-	
V119	1745	NI	<0.1	<0.1	0.86 ± 0.021	0.76 ± 0.021	-	-	-	
V120	1751	NI	0.48 ± 0.007	0.67 ± 0.021	0.52 ± 0.014	0.69 ± 0.007	-	-	-	
V121	1764	Guaiol acetate	-	-	-	-	-	0.28 ± 0.057	0.47 ± 0.028	OS
V122	1806	NI	-	-	-	-	-	1.2 ± 0.071	1.8 ± 0.134	
Hydrocarbon monoterpene			7.6	8.3	0.6	4.1	28.2	0.6	0.5	HM
Hydrocarbon sesquiterpene			39.5	29.9	39.9	31.7	65.1	15.6	12.4	HS
Ketone			5.0	5.1	1.9	2.6	0.0	0.0	0.0	Ke
Oxygenated monoterpene			12.6	15.3	2.9	5.4	1.7	11.3	10.9	OM
Oxygenated sesquiterpene			5.1	6.9	14.9	15.8	3.8	11.8	9.7	OS
Phenylpropanoid			-	-	-	-	-	56.7	62.0	Ph
Not identified (unknown)			26.7	29.4	36.4	35.6	0.9	3.7	4.0	NI
		Total (%)	96.08	94.45	96.28	94.90	99.60	99.75	99.55	
		Yield (*v*/*w* %)	0.91	0.91	0.93	1.2	3.6	0.6	0.5	

* IR—retention index calculated; NI—not identified (unknown); **: Chemical Class; Le-F-1—leaves from the flooding season of chemotype-1; Le-D-1—leaves from the dry season of chemotype-1; Le-F-2—leaves from the flooding season of chemotype-2; Le-D-2—leaves from the dry season of chemotype-2; Ba-D-1—bark from the dry season of chemotype-1; Ba-F-1—bark from the flooding season of chemotype-1; Fr-1—fruits from the flooding season of chemotype-1. Metabolites highlighted in bold represent the major compound of each plant organ.

## Data Availability

The molecular network is available at the following link https://gnps.ucsd.edu/ProteoSAFe/status.jsp?task=3892fb335b6d47b2a2f76a0631165574 (accessed on 23 April 2025).

## Supplementary Materials

The following supporting information can be downloaded at: https://www.mdpi.com/article/10.3390/plants14131939/s1, Table S1: Information about the 14 individuals of *Mespilodaphne cymbarum* collected in the Mamirauá Sustainable Development Reserve. Figure S1: Heatmap and Hierarchical Clustering Analysis (HCA) of the volatiles extracted by SPME from the leaves of 14 individuals of *M. cymbarum*, obtained in the flooding (F) and dry (D) seasons. Figure S2: Heatmap and Hierarchical Clustering Analysis (HCA) of the volatiles from the essential oils (EOs) extracted by hydrodistillation from the leaves of *M. cymbarum.* Figure S3: Molecular network of the essential oils extracted from the leaves, bark and fruits of *M. cymbarum*. Figure S4: Antimicrobial and antibiofilm activity of the essential oils (EOs) from leaves, bark and fruits of *M. cymbarum* against three Gram-negative bacterial strains.

## Abbreviations

The following abbreviations are used in this manuscript:

EO/EOs	Essential oil/Essential oils
GC-MS	Gas chromatography–mass spectrometry
DMSO	Dimethyl sulfoxide
Le-D-1	Leaves from chemotype-1 collected in the dry season
Le-F-1	Leaves from chemotype-1 collected in the flooding season
Le-D-2	Leaves from chemotype-2 collected in the dry season
Le-F-2	Leaves from chemotype-2 collected in the flooding season
Ba-D-1	Bark from chemotype-1 collected in the dry season
Ba-F-1	Bark from chemotype-1 collected in the flooding season
Fr-1	Fruit from chemotype-1 collected in the flooding season
ATCC	American Type Culture Collection
PAO1	*Pseudomonas aeruginosa* resistant to chloramphenicol
