# Supplementary material for: Chemotypic and Seasonal Variations in Essential Oils from Mespilodaphne cymbarum (Kunth) Trofimov and Their Antibacterial and Antibiofilm Activities
Source: Plants (Basel). 2025 Jun 24;14(13):1939. doi: 10.3390/plants14131939 (PMC12252200; doi:10.3390/plants14131939)
Supplement: Supplementary file 1 [file plants-14-01939-s001.zip › plants-3681580-supplementary.pdf]

plants

MDPI

Supplementary materials - Supporting Information

Article

Chemotypic and Seasonal Variations in Essential Oils from *Mespilodaphne cymbarum* (Kunth) Trofimov and Their Antibacterial and Antibiofilm Activities

Amanda Galdi Boaretto [1,2], Darlene Gris [3], Jéssica Scherer [4], Katyuce Souza Farias [2], Jean Carlo Quadros [3], Alexandre José Macedo [4], Carlos Alexandre Carollo [2] and Denise Brentan Silva [2,*]

[1] , Instituto de Biociências, Universidade Federal de Mato Grosso do Sul, Cidade Universitária, Campo Grande 79070-900, Mato Grosso do Sul, Brazil; amanda.boaretto@ufms.br
[2] Laboratório de Produtos Naturais e Espectrometria de Massas (LaPNEM), Faculdade de Ciências Farmacêuticas, Alimentação e Nutrição (FACFAN), Universidade Federal de Mato Grosso do Sul, Cidade Universitária, Campo Grande 79070-900, Mato Grosso do Sul, Brazil; katyuce.farias@gmail.com (K.S.F.); carlos.carollo@ufms.br (C.A.C.)
[3] Instituto de Desenvolvimento Sustentável Mamirauá, Estrada do Bexiga, Tefé 69553-225, Amazonas, Brazil; darlene.gris@mamiraua.org.br (D.G.);
jeancdq@gmail.com (J.C.d.Q.)
[4] Laboratório de Biofilmes e Diversidade Microbiana, Faculdade de Farmácia e Centro de Biotecnologia, Universidade Federal do Rio Grande do Sul, Porto Alegre 91501-970, Rio Grande do Sul, Brazil; jessicscherer@gmail.com (J.S.); alexandre.jose.macedo@gmail.com (A.J.M.)
* Correspondence: denise.brentan@ufms.br; Tel.: +55-67-3345-7366

SPME extraction of the volatiles from leaves of Mespilodaphne cymbarum

Volatile organic compounds (VOCs) from leaves were extracted using a solid-phase microextraction (SPME) fiber from SUPELCO, consisting of 100 μm polydimethylsiloxane (PDMS) and fused silica (24 Ga) using a manual holder. Dried and powdered leaves (60 mg) from each individual were placed in a 4 mL sealed vial with polytetrafluoroethylene (PTFE) lined caps. Each vial was incubated in a water bath at 40 °C, after which the SPME fiber inserted and exposed for 50 min to adsorb the volatiles. Subsequently, this fiber was inserted into the gas chromatography–mass spectrometry (GC-MS) system for analysis, under the same condition presented in material and methods.

The GC-MS data was aligned in the MetAlign 3.0 software and the entrances were reduced using MSClust. A pooled sample, prepared by mixing all individuals collected from both seasons, was used as a reference for the alignment and served as quality control of the analysis. The resulting intensity matrix was used for statistical analysis in Metaboanalyst 6.0. The data were log-transformed and autoscaled to normalize the variables and make them comparable. Hierarchical Clustering Analysis (HCA) and a heatmap were generated selecting the top 50 metabolites, revealing two distinct chemotypes (Figure S1). This result guided the decision on how to extract the essential oils (EOs) in this article. Besides, when comparing the 14 specimens, no clear distinction was observed between samples collected during the flooding and dry seasons.

Table S1. Information about the 14 individuals of *Mespilodaphne cymbarum* collected in the Mamirauá Sustainable Development Reserve, Uarini, AM, Brazil.

Ind.*	DBH** (cm)	Altitude (m)	Latitude	Longitude	Date - dry	Date - flooding	Chemotypes
**14**	234	121	-2.833757	-65.039765	06/09/2021	13/04/2022	Chemo-1
**26**	297	127	-2.829916	-65.046491	06/09/2021	13/04/2022	Chemo-1
**31**	156	131	-2.827673	-65.05038	06/09/2021	13/04/2022	Chemo-1
**42**	205	137	-2.822524	-65.057377	06/09/2021	13/04/2022	Chemo-2
**44**	44	139	-2.821709	-65.061737	07/09/2021	14/04/2022	Chemo-1
**45**	232	140	-2.822117	-65.059984	07/09/2021	14/04/2022	Chemo-1
**55**	155	149	-2.828608	-65.050539	07/09/2021	14/04/2022	Chemo-1
**61**	124	154	-2.833756	-65.040921	07/09/2021	14/04/2022	Chemo-1
**71**	66	163	-2.84599	-64.989552	07/09/2021	14/04/2022	Chemo-2
**73**	300	165	-2.857055	-64.98876	07/09/2021	14/04/2022	Chemo-1
**82**	103	173	-2.8148	-65.077723	06/09/2021	13/04/2022	Chemo-1
**85**	154	176	-2.809894	-65.090945	06/09/2021	13/04/2022	Chemo-1
**87**	213	178	-2.806041	-65.094782	06/09/2021	13/04/2022	Chemo-1
**95**	108	186	-2.814598	-65.082174	06/09/2021	13/04/2022	Chemo-2

*Ind. – Individuals (the number codifying the individuals were the identifications used in the field); **Diameter at breast height (DBH); Latitude and longitude are given in decimal degrees.

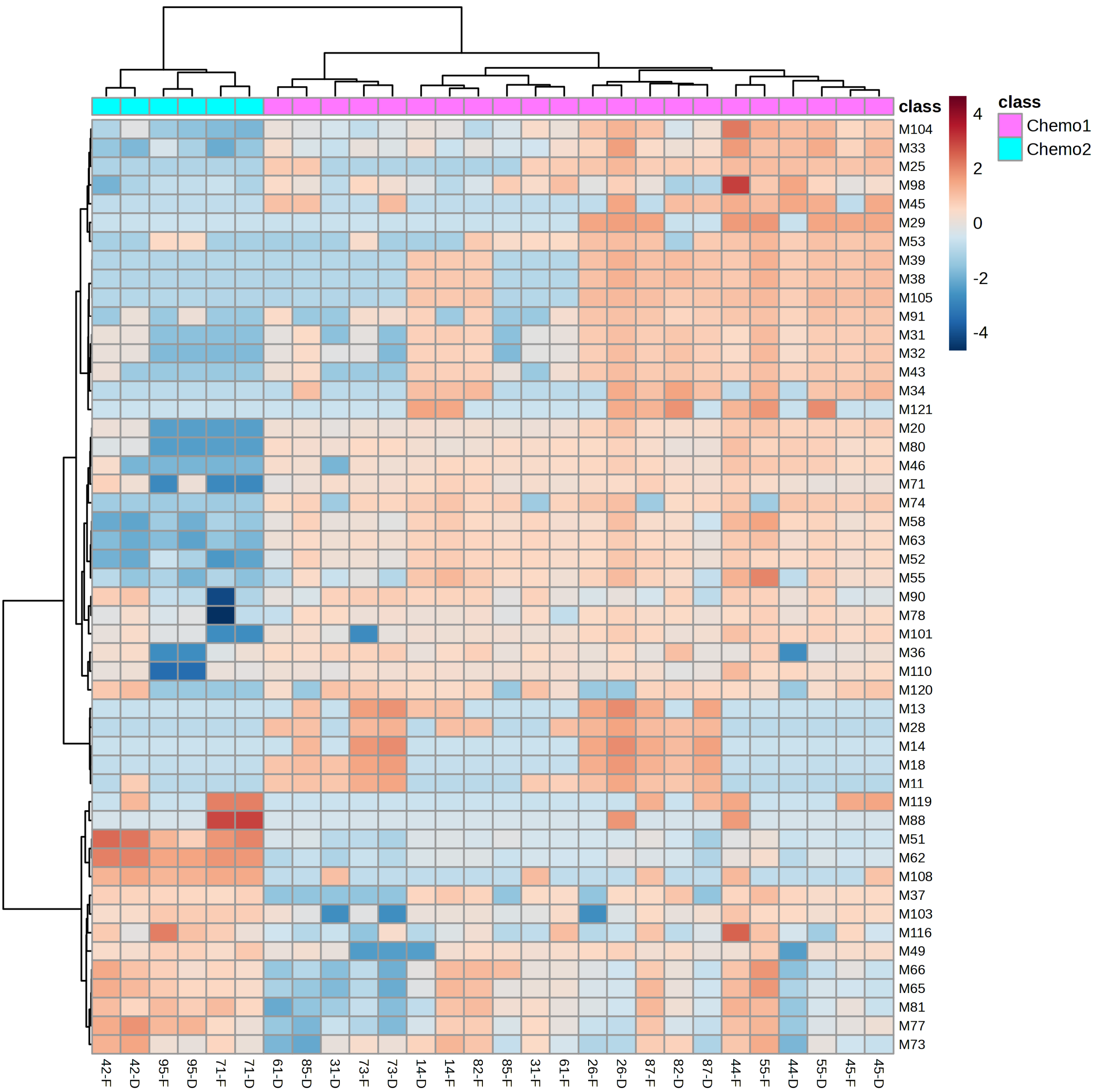

Figure S1. Heatmap and Hierarchical Clustering Analysis (HCA) of the top 50 volatiles extracted by SPME from the leaves of 14 individuals of *Mespilodaphne cymbarum*, obtained in the flooding (F) and dry (D) seasons. Chemotype-1 (pink) grouping eleven individuals, and chemotype-2 (blue) is composed of three individuals.

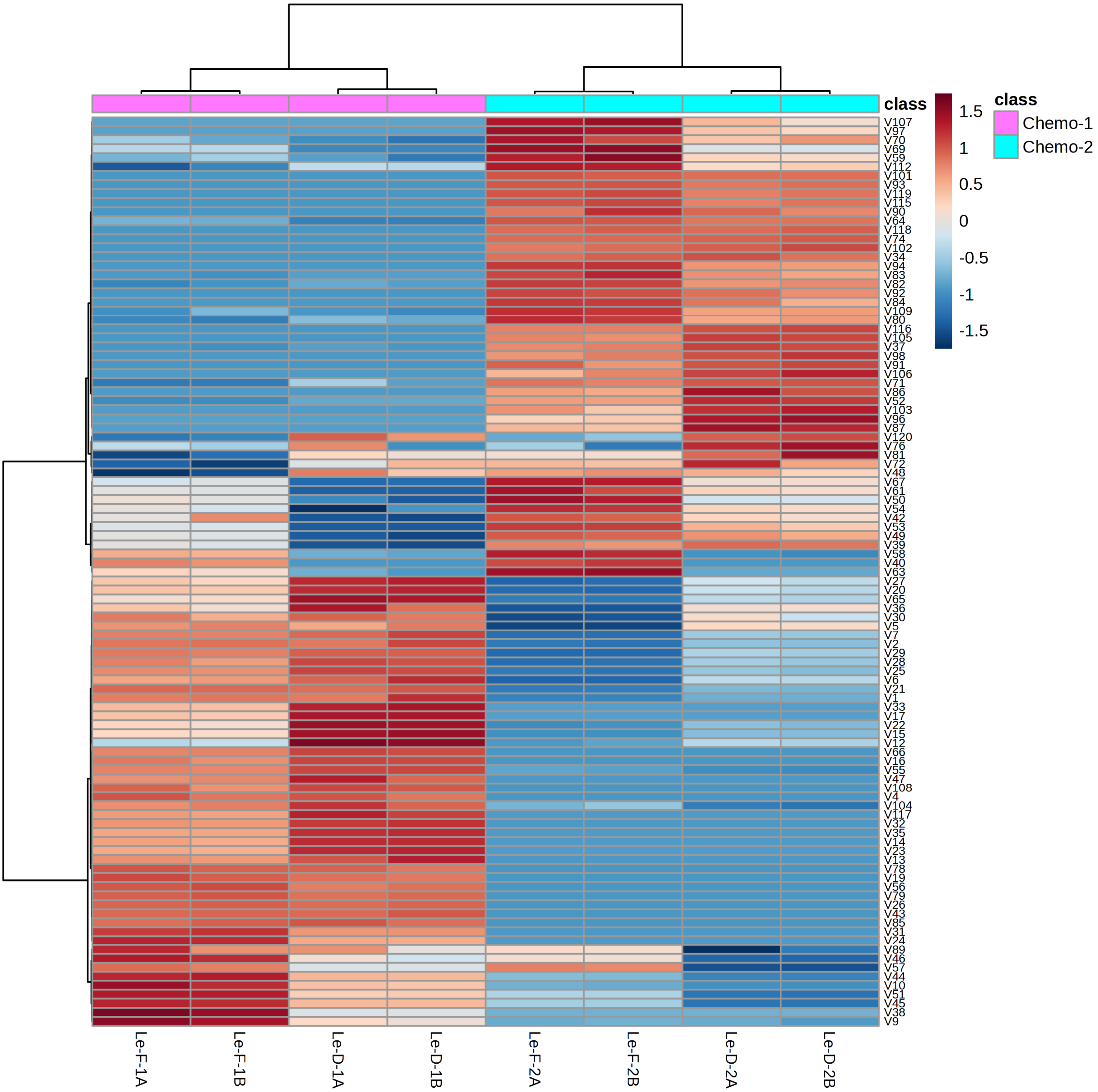

Figure S2: Heatmap and Hierarchical Clustering Analysis (HCA) of the volatiles from the essential oils (EOs) extracted by hydrodistillation from the leaves of *Mespilodaphne cymbarum.* Chemotype-1 (pink) and chemotype-2 (blue) formed two distinct clusters. **Le-F-1** - leaves from the flooding season of chemotype-1; **Le-D-1** – leaves from the dry season of chemotype-1; **Le-F-2** - leaves from the flooding season of chemotype-2; **Le-D-2** – leaves from the dry season of chemotype-2. The letters A and B represent the analytical replicates from each season.

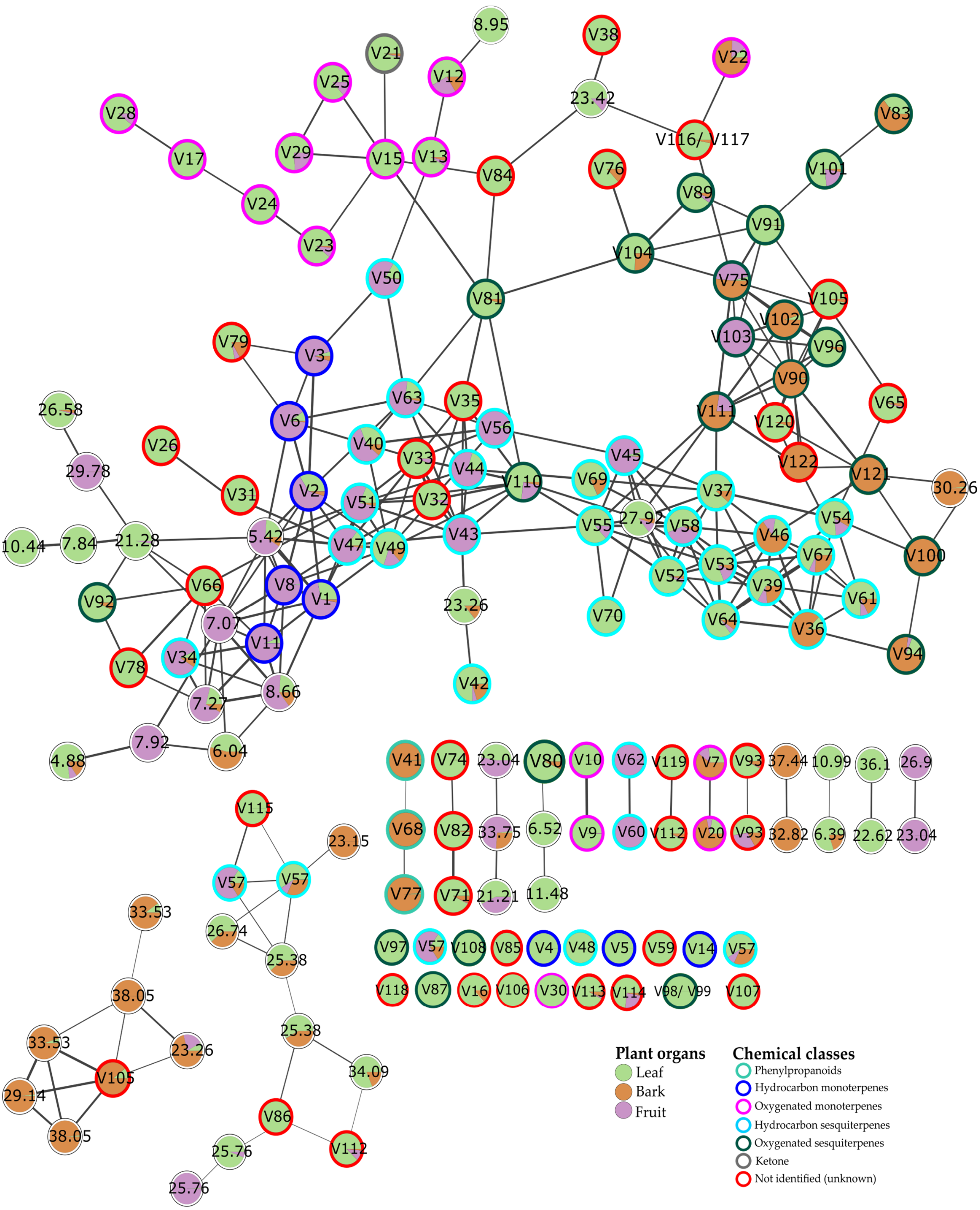

Figure S3: Molecular network of the essential oils extracted from the leaves, bark and fruits of *Mespilodaphne cymbarum*. Nodes labeled by retention time (min) correspond to low-intensity signals in our analysis and were therefore excluded from the main compound identification table. This data is available at (https://gnps.ucsd.edu/ProteoSAFe/status.jsp?task=3892fb335b6d47b2a2f76a0631165574).

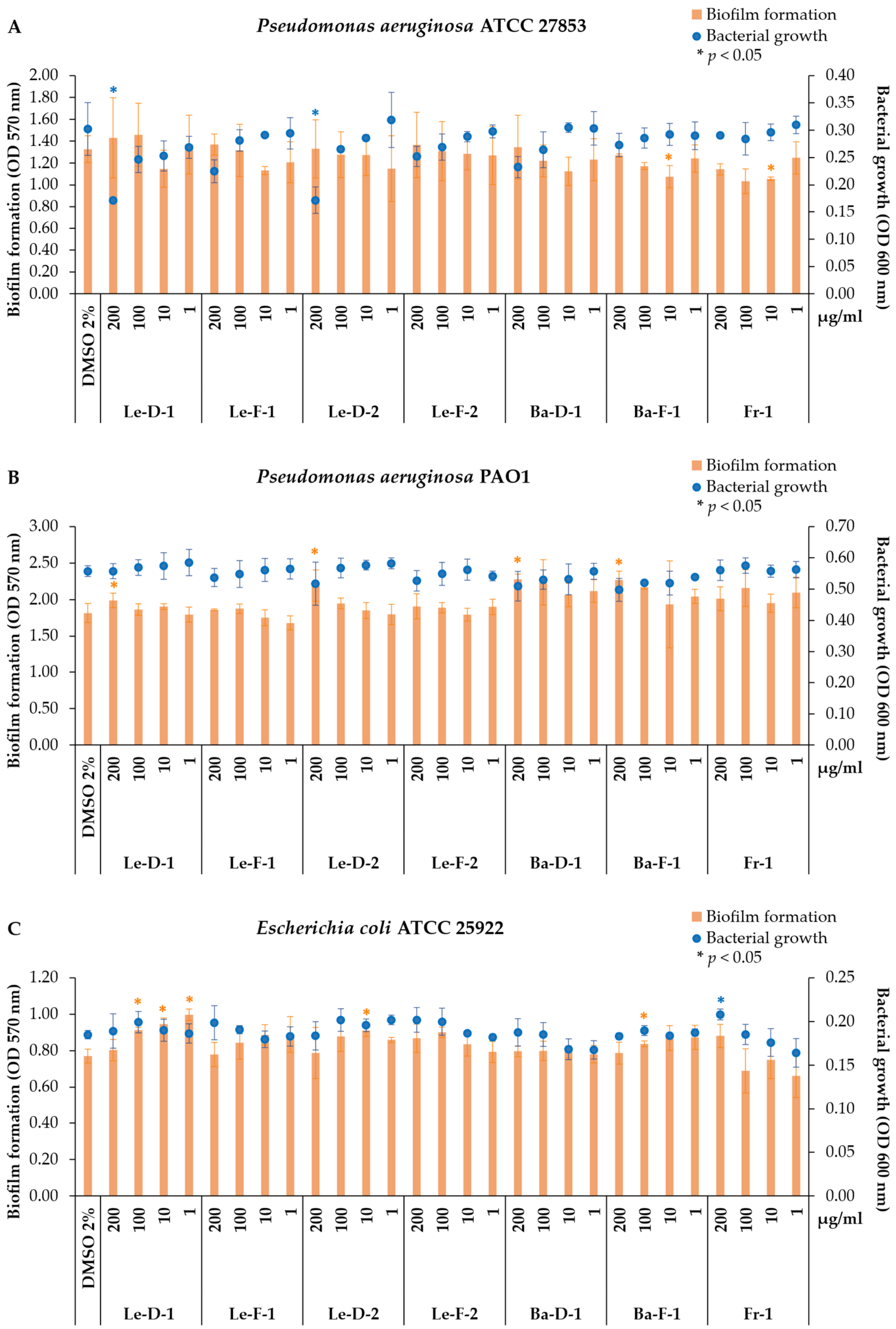

Figure S4: Antimicrobial and antibiofilm activity of the essential oils (EOs) from leaves, bark and fruits of *Mespilodaphne cymbarum* against three Gram-negative bacterial strains: (**A**) *Pseudomonas aeruginosa* ATCC 27853, (**B**) *Pseudomonas aeruginosa* PAO1, and (**C**) *Escherichia coli*. Control treatment contain dimethyl sulfoxide 2% (DMSO 2%); treatment with the essential oils (EOs): Le-D-1 (leaves from the dry season of chemotype-1); Le-F-1 (leaves from the flooding season of chemotype-1); Le-D-2 (leaves from the dry season of chemotype-2); Le-F-2 (leaves from the flooding season of chemotype-2); Ba-D-1 (bark from the dry season of chemotype-1); Ba-F-1 (bark from the flooding season of chemotype-1); Fr-1 (fruits from the flooding season of chemotype-1). (*) Significant activity compared with the control (DMSO 2%) by paired *t*-test (*$p < 0.05$). Positive control used was meropenem 8 µg/mL.
